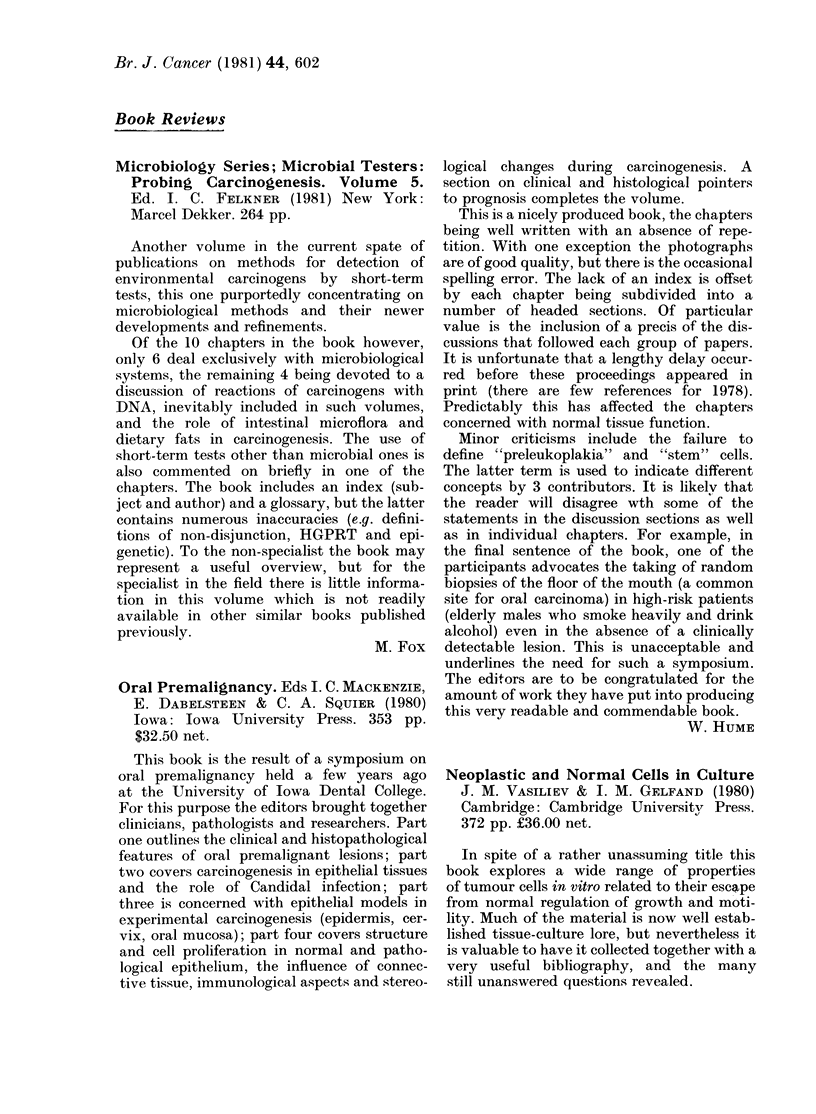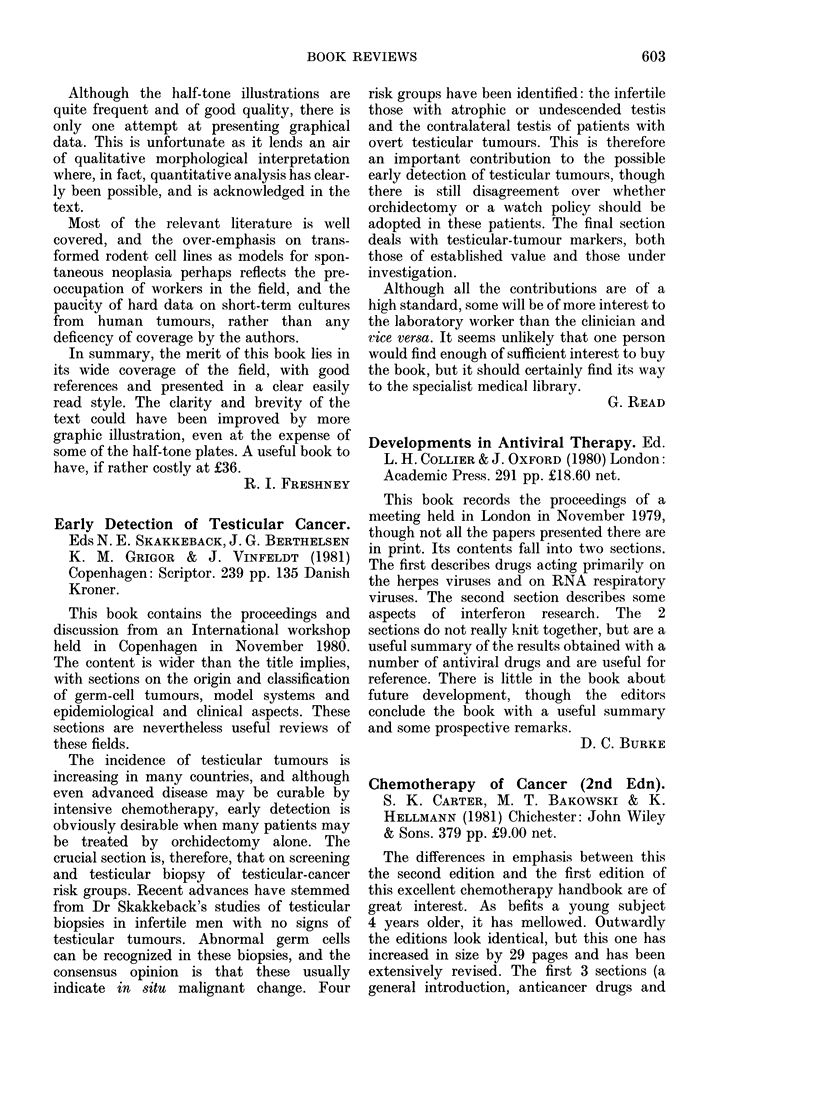# Neoplastic and Normal Cells in Culture

**Published:** 1981-10

**Authors:** R. I. Freshney


**Neoplastic and Normal Cells in Culture** J. M. Vasiliev & I. M. Gelfand (1980) Cambridge: Cambridge University Press. 372 pp. £36.00 net.

In spite of a rather unassuming title this book explores a wide range of properties of tumour cells *in vitro* related to their escape from normal regulation of growth and motility. Much of the material is now well established tissue-culture lore, but nevertheless it is valuable to have it collected together with a very useful bibliography, and the many still unanswered questions revealed.

Although the half-tone illustrations are quite frequent and of good quality, there is only one attempt at presenting graphical data. This is unfortunate as it lends an air of qualitative morphological interpretation where, in fact, quantitative analysis has clearly been possible, and is acknowledged in the text.

Most of the relevant literature is well covered, and the over-emphasis on transformed rodent cell lines as models for spontaneous neoplasia perhaps reflects the preoccupation of workers in the field, and the paucity of hard data on short-term cultures from human tumours, rather than any deficency of coverage by the authors.

In summary, the merit of this book lies in its wide coverage of the field, with good references and presented in a clear easily read style. The clarity and brevity of the text could have been improved by more graphic illustration, even at the expense of some of the half-tone plates. A useful book to have, if rather costly at £36.

R. I. FRESHNEY